# Iron Metabolism, Pseudohypha Production, and Biofilm Formation through a Multicopper Oxidase in the Human-Pathogenic Fungus Candida parapsilosis

**DOI:** 10.1128/mSphere.00227-20

**Published:** 2020-05-13

**Authors:** Tanmoy Chakraborty, Zsófia Tóth, Renáta Tóth, Csaba Vágvölgyi, Attila Gácser

**Affiliations:** a Department of Microbiology, University of Szeged, Szeged, Hungary; b MTA-SZTE Lendület Mycobiome Research Group, University of Szeged, Szeged, Hungary; University of Georgia

**Keywords:** *Candida parapsilosis*, biofilms, fungal multicopper oxidase, pseudohypha

## Abstract

Among all the essential micronutrients, iron plays an important role in mammalian biology. It is also essential for pathogens infecting mammalian hosts, including bacteria, fungi, and protozoans. As the availability of accessible iron is limited within the mammalian host, several human-pathogenic fungal pathogens, such as Candida albicans, Cryptococcus neoformans, Candida glabrata, and Aspergillus fumigatus, have developed various iron uptake mechanisms. Although Candida parapsilosis is the second or third most common non-*albicans Candida* species associated with systemic and superficial *Candida* infections in immunocompromised patients, the mechanisms of iron uptake and homoeostasis remain unknown in this fungus. In the current report, we show that a homologue of the multicopper oxidase gene *FET3* is present in the genome of C. parapsilosis (*CPAR2_603600*) and plays a significant role in iron acquisition. We found that homozygous deletion mutants of *CPAR2_603600* showed defects under low-iron conditions and were also sensitive to various stressors. Our results also revealed that the levels of pseudohypha formation and biofilm formation were reduced in the null mutants compared to the wild type. This phenotypic defect could be partially rescued by supplementation with excess iron in the growth medium. The expression levels of the orthologues of various iron metabolism-related genes were also altered in the mutants compared to the parental strain. In conclusion, our report describes the role of *CPAR2_603600* in iron homoeostasis maintenance as well as morphology and biofilm formation regulation in this pathogenic fungus.

**IMPORTANCE** C. parapsilosis is the second or third most common opportunistic human-pathogenic *Candida* species, being responsible for severe fungal infections among immunocompromised patients, especially low-birth-weight infants (0 to 2 years of age). Among the major virulence factors that pathogenic fungi possess is the ability to compete with the host for essential micronutrients, including iron. Accessible iron is required for the maintenance of several metabolic processes. In order to obtain accessible iron from the host, pathogenic fungi have developed several iron acquisition and metabolic mechanisms. Although C. parapsilosis is a frequent cause of invasive candidiasis, little is known about what iron metabolic processes this fungus possesses that could contribute to the species’ virulent behavior. In this study, we identified the multicopper oxidase *FET3* gene that regulates iron homeostasis maintenance and also plays important roles in the morphology of the fungus as well as in biofilm formation, two additional factors in fungal virulence.

## Abstract (precis)

C. parapsilosis is the second or third most common opportunistic human-pathogenic *Candida* species, being responsible for severe fungal infections among immunocompromised patients, especially low-birth-weight infants (0 to 2 years of age). Among the major virulence factors that pathogenic fungi possess is the ability to compete with the host for essential micronutrients, including iron. Accessible iron is required for the maintenance of several metabolic processes. In order to obtain accessible iron from the host, pathogenic fungi have developed several iron acquisition and metabolic mechanisms. Although C. parapsilosis is a frequent cause of invasive candidiasis, little is known about what iron metabolic processes this fungus possesses that could contribute to the species’ virulent behavior. In this study, we identified the multicopper oxidase *FET3* gene that regulates iron homeostasis maintenance and also plays important roles in the morphology of the fungus as well as in biofilm formation, two additional factors in fungal virulence.

## INTRODUCTION

Iron is one of the most important micronutrients necessary for the growth and propagation of pathogens during an infection. It plays a crucial role in the structure of many proteins and in their enzymatic functions. Generally, it is incorporated into a heme complex or bound into iron sulfur clusters. Iron also acts as an important cofactor in different cellular processes such as the tricarboxylic acid (TCA) cycle, DNA replication, chromatin remodeling, mitochondrial respiration, and detoxification of reactive oxygen species (ROS) (1). Iron is a transition element which can exist in two oxidation states: a reduced ferrous (Fe^2+^) form or a oxidized ferric (Fe^3+^) form. The capacity of iron to gain or lose electrons is the reason why iron is the most important redox mediator in biology. Also, the divalent form of iron (Fe^2+^) has the potential to catalyze the formation of cell-damaging hydroxyl radicals via the Fenton/Haber Weiss reaction (2).

In Saccharomyces cerevisiae, the Fet3 protein is required for high-affinity iron transport. The protein works downstream of the ferric reductases and is a cell surface ferroxidase belonging to the multicopper oxidase family (3). From expression analysis of *FET3*, it has been shown that it is regulated by the transcription factor Aft1p and the iron concentration of the environment. The mRNA transcripts of the genes *FRE1*, *FRE2*, and *FET3* are not detected in the absence of a functional *AFT1* gene (4). *FET3* mRNA levels were increased after growth in low-iron (1 to 10 μM) medium, whereas expression levels were below the detectable range when cells were grown in high-iron (1,000 μM) medium (5).

The crucial role of iron uptake and metabolism in human-pathogenic fungi such as Candida albicans, Cryptococcus neoformans, and Aspergillus fumigatus is well known, and the role of iron during opportunistic fungal infections has been extensively studied (6, 7). As *Candida* spp. belong to the normal human microbiota, various iron acquisition and metabolic mechanisms have evolved due to the hardships impeding accessible iron uptake. Pathways involved in C. albicans and C. glabrata iron metabolism have already been examined (8–11). Several genes have been revealed as necessary for reduction of ferric to ferrous iron and also for the uptake of reductive iron. The three main steps of reductive iron assimilation are (i) reduction of extracellular Fe^3+^ to Fe^2+^, mediated by surface-bound ferric reductases; (ii) reoxidation of Fe^2+^ to Fe^3+^ by multicopper ferroxidases; and (iii) Fe^3+^ import by the permease Ftr1. Notably, *FET3* is required for the growth of C. albicans under iron-limiting conditions but not for virulence (12). Also, C. albicans has a transcription circuit for iron metabolism that enables survival within the human gastrointestinal tract as a commensal organism (13).

C. parapsilosis, a member of the CTG clade, is a major cause of neonatal candidiasis and also affects other patients with an immunocompromised status (14, 15). The hands of health care workers are often colonized by C. parapsilosis, which is the most relevant source of transmission and outbreaks in hospitals (16). There are various virulence factors that contribute to the pathogenicity of this species. These include adhesion to various biotic and abiotic surfaces, hydrolytic enzyme production, pseudohypha production, biofilm production on catheters and other implanted devices, eicosanoid production, and resistance to different classes of antifungal drugs (17). Although iron metabolism and its role in the virulence of the closely related species C. albicans have been well studied, little is known about iron homeostasis regulation and its relevance in the virulence of C. parapsilosis. Previously, it was reported that *CPAR2_100540*, a gene orthologous to C. albicans *HAP5* (*CaHAP5*), regulates iron metabolism as well as virulence in this species (18). Although *FET3* is not linked to virulence in C. albicans, we recently demonstrated that C. parapsilosis *FET3* (*CpFET3*), besides playing a role in prostaglandin production, influences its virulence (19); however, its role in iron metabolism remains unclear. In our current study, we showed that *CpFET3* is also involved in the regulation of iron metabolism and also influences fungal cell morphology and biofilm formation.

## RESULTS

### Identification of three multicopper oxidase genes in C. parapsilosis by *in silico* analysis and their expression under iron-limiting conditions.

**FIG 1:**
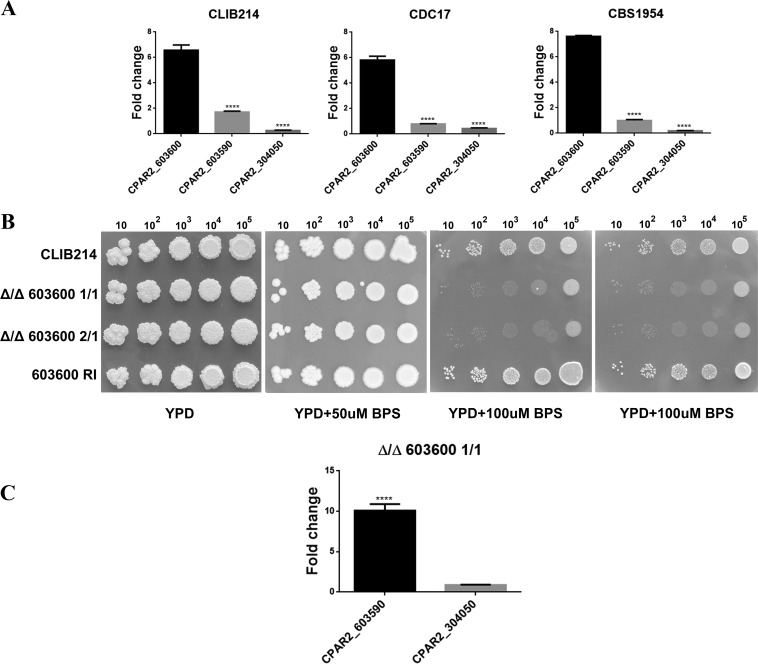
Expression analysis of multicopper oxidase-encoding genes and the effect of *CPAR2_603600* deletion on growth. (A) Expression analysis of *CPAR2_603600*, *CPAR2_304050*, and *CPAR2_603590* in CLIB 214, CDC17, and CBS1954 C. parapsilosis isolates under low-iron conditions. Fold change expression was calculated taking YPD growth as the control condition. Statistical significance was calculated using one-way ANOVA coupled with Dunnett’s *post hoc* test (****, *P* ≤ 0.0001). (B) Growth analysis of CLIB 214 parental, Δ/Δ*603600 1*/*1*, Δ/Δ*603600 2*/*1*, and *603600 RI* strains in the absence and presence of the iron chelator BPS in increasing concentrations. Images were taken after 3 days of incubation at 30°C. (C) Expression of *CPAR2_603590* and *CPAR2_304050* in the absence of *CPAR2_603600.* Statistical significance was calculated using the test mentioned above (****, *P* ≤ 0.0001).

To identify multicopper oxidases in C. parapsilosis, a BLAST search was performed (www.candidagenome.org) (20) using Saccharomyces cerevisiae Fet3p (YMR058W) as the query sequence. We identified only three genes in the multicopper oxidase family with >40% identity to the query sequence, which is in contrast with the identification of the five *FET3* orthologs *FET3*, *FET31*, *FET33*, *FET34*, and *FET99* in analyses of C. albicans (see Fig. S2 in the supplemental material) (8). The three genes identified were *CPAR2_603600*, *CPAR2_304050*, and *CPAR2_603590* (Fig. S1). The results of multiple-sequence alignment (21, 22) showed that CPAR2_603600p, CPAR2_603590p, and CPAR2_304050p have 79%, 75.3%, and 66% sequence identity with *Ca*Fet3p, *Ca*Fet99p, and *Ca*Fet33p, respectively (Fig. S3).To examine if these genes might be associated with iron metabolic processes, we assessed their expression levels under iron-limited conditions using three different C. parapsilosis isolates: CLIB214, CBS1954 (23), and CDC17. For growth, 100 μM/ml bathophenanthroline disulfonate (BPS)-supplemented YPD (1% dextrose, 1% peptone, and 0.5% yeast extract) liquid medium was used. As a control, cells were also grown without the addition of BPS. According to our results, the expression of *CPAR2_603600* was significantly elevated in all three isolates compared to that of the other two genes, suggesting its greater influence under iron-limited growth conditions (Fig. 1A).

10.1128/mSphere.00227-20.1 FIG S1 Multiple-sequence alignment of *Sc*Fet3p with CPAR2_603600p, CPAR2_603590p, and CPAR2_304050p. Download FIG S1, TIF file, 0.5 MB. Copyright © 2020 Chakraborty et al. 2020 Chakraborty et al. This content is distributed under the terms of the Creative Commons Attribution 4.0 International license.

10.1128/mSphere.00227-20.2 FIG S2 Homology between *Sc*Fet3p and five multicopper oxidase proteins in C. albicans. Download FIG S2, TIF file, 1.4 MB. Copyright © 2020 Chakraborty et al. 2020 Chakraborty et al. This content is distributed under the terms of the Creative Commons Attribution 4.0 International license.

10.1128/mSphere.00227-20.3 FIG S3 Similarity between amino acid sequences of CPAR2_603600p and *Ca*Fet3p, of CPAR2_304050p and *Ca*Fet33p, and of CPAR2_603590p and *Ca*Fet99p. Download FIG S3, TIF file, 1.3 MB. Copyright © 2020 Chakraborty et al. 2020 Chakraborty et al. This content is distributed under the terms of the Creative Commons Attribution 4.0 International license.

### *CPAR2_603600* regulates growth under low-iron conditions.

To thoroughly investigate the role of *CPAR2_603600* in iron metabolism, two independent homozygous deletion mutants (the Δ/Δ*603600 1*/*1* and Δ/Δ*603600 2*/*1* mutants) were generated using C. parapsilosis isolate CLIB214 as a parental strain. Gene deletion was performed using the fusion PCR-based auxotrophy complementation method previously described by Holland et al. (24). For all the subsequent analyses, a *CPAR2_603600* reintegrant (RI) mutant strain was also applied. To examine the effect of gene deletion on growth under conditions of iron starvation, parental, reintegrant, and null mutant strains were spotted on YPD plates supplemented with 50, 100, and 150 μg/ml BPS, chelating ferrous iron (II). Nonsupplemented YPD medium was also used as a control. Although all analyzed strains grew similarly on YPD plates, the Δ/Δ*603600 1*/*1* and Δ/Δ*603600 2*/*1* strains showed a remarkable growth deficiency under iron-limited conditions (>100 μg/ml BPS) compared to the wild-type (WT) strain and the RI strains, suggesting that *CPAR2_603600* is required for viability only under low-iron conditions (Fig. 1B). We also analyzed the expression of *CPAR2_304050* and *CPAR2_603590* in the absence of *CPAR2_603600*. As shown in Fig. 1C, expression of *CPAR2_603590* increased remarkably in the deletion mutant strain compared to the parental strain but that of *CPAR2_304050* did not (Fig. 1A), suggesting a potential compensatory effect in the case of *CPAR2_603590*.

### The Δ/Δ*603600* mutant showed growth defects under stress-inducing conditions.

**FIG 2:**
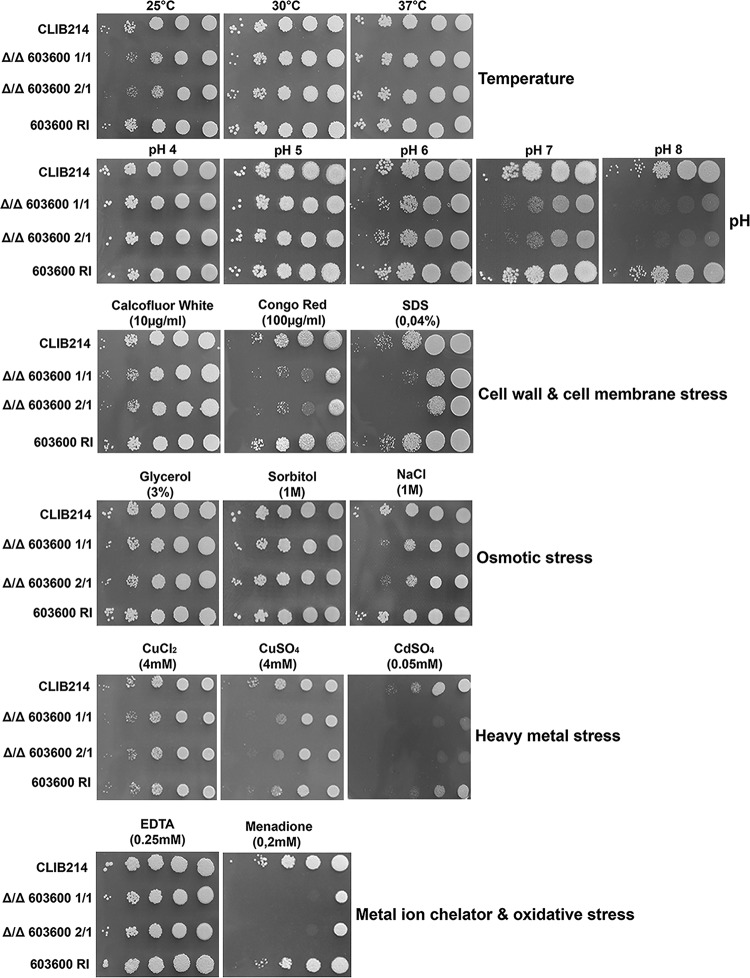
Phenotypic characterization of the mutant under different growth conditions. Results of phenotypic screening of the CLIB parental strain, Δ/Δ*603600* homozygous deletion mutants, and the *603600 RI* strain under various growth conditions, including different temperatures and pH levels and YPD medium supplemented with osmotic stressors, cell wall stressors, cell membrane stressors, oxidative stressors, or heavy-metal stressors, are shown. Different dilutions of cells were spotted on each plate, and pictures were taken after 2 days of growth at 30°C.

Phenotypic characterization of the deletion mutants was performed by growth analyses under 19 different growth conditions, including different temperatures, different pH levels, and the presence or absence of cell wall stressors, cell membrane stressors, osmotic stressors, oxidative stressors, and heavy-metal stressors (Fig. 2). The mutants showed a growth-deficient phenotype at lower temperature (25°C) and alkaline pH in the presence of the cell wall stressor Congo red, a cell membrane stressor (SDS), an oxidative stressor (menadione), and a heavy-metal stressor (cadmium sulfate), whereas the mutants were less sensitive to heavy-metal copper. There were no differences in the levels of growth in the presence of osmotic stress created by sorbitol or glycerol, although null mutant strains showed a slight growth defect in response to NaCl.

### *CPAR2_603600* influences morphology transition.

**FIG 3:**
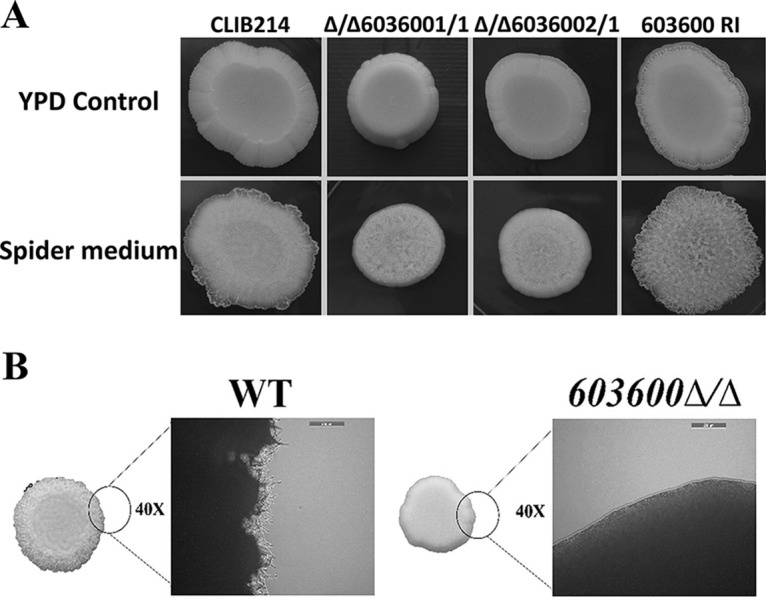
Decrease in pseudohypha formation in the absence of *CPAR2_603600.* (A) Colony morphology of the *CPAR2_603600* mutant and wild-type strains was analyzed in pseudohypha-inducing medium (Spider medium) maintained at 37°C after 7 days. The YPD plate served as a control. (B) Microscopic images of the edge of wild-type (WT) and Δ/Δ*603600 1*/*1* colonies on solid Spider medium (scale bar, 160 μm).

**FIG 4:**
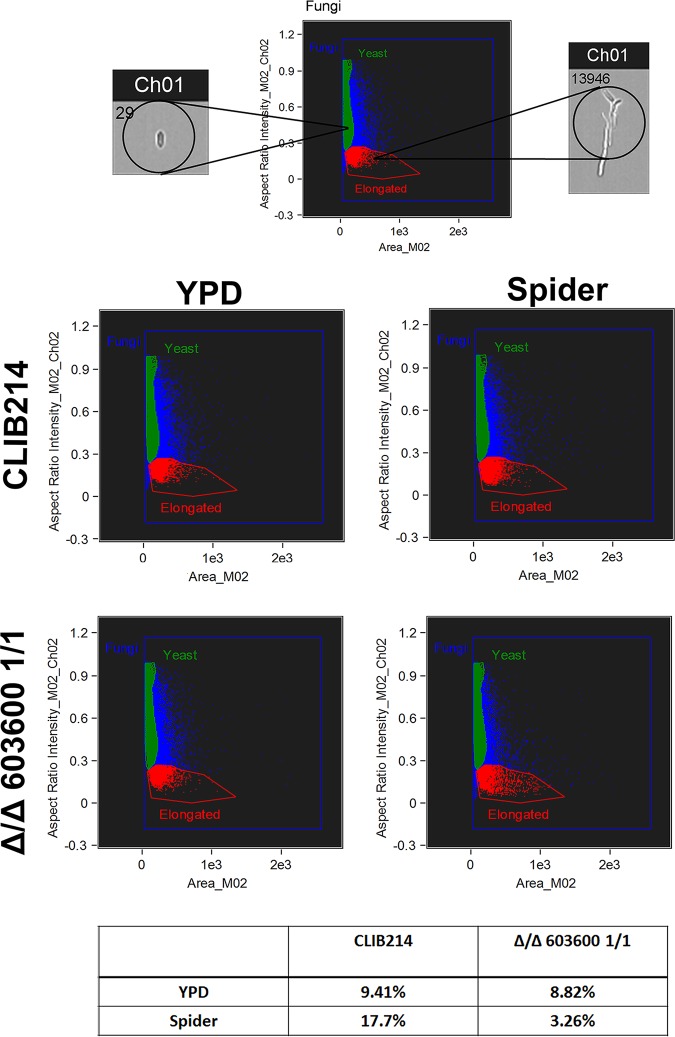
Percentage of pseudohypha formation by FACS analysis. Pseudohypha formation was determined by quantitative FACS analysis after growth in YPD and pseudohypha-inducing media at 37°C in the presence of 5% CO_2_.

**FIG 5:**
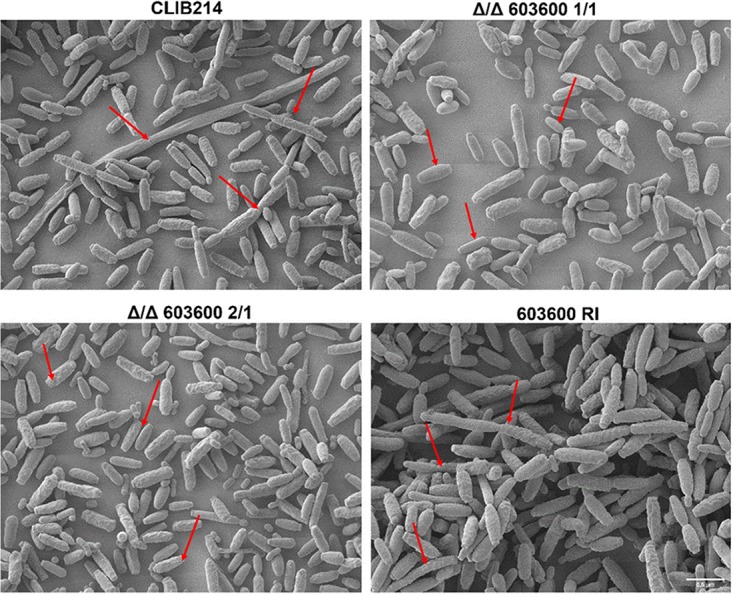
Scanning electron microscopic images of the wild-type and CPAR2_603600 mutant strains. All strains were visualized using SEM after growth in Spider medium at 37°C for 24 h. Arrows indicate greater numbers of elongated pseudohyphal cells in wild-type and reintegrant strains than in the null mutants, where most of the yeast cells were smaller.

One of the most important virulence traits of C. parapsilosis is its ability to produce pseudohyphae. In C. albicans, two iron metabolism regulatory genes, *TUP1* and *FET34*, regulate filamentous growth (25, 26). Thus, we examined whether *CPAR2_603600* has a similar effect in C. parapsilosis. Interestingly, deletion of *CPAR2_603600* resulted in a significant reduction in pseudohypha formation both on solid medium and in liquid medium compared to the wild-type strain and the reintegrant strains (Fig. 3). A difference in colony wrinkling between the wild-type strain and the mutant strains was detected on solid Spider medium after 7 days of incubation at 37°C (Fig. 3A). Microscopic pictures of the colony edges on Spider medium revealed similar differences and thus reduced pseudohypha formation of the Δ/Δ*603600 1*/*1* mutant compared to the parental strain (Fig. 3B). Single-colony analysis of the wild-type strain and the Δ/Δ*603600 1*/*1* strain on Spider medium also showed a difference in colony morphology (Fig. S4). Quantitative analysis of pseudohypha formation was also performed using liquid medium (Fig. 4). In liquid Spider medium, pseudohypha formation was enhanced (for the wild-type strain, 9.41% in YPD control medium versus 17.7% in Spider medium). Furthermore, under the same condition, the Δ/Δ*603600 1*/*1* mutant strain showed a significant reduction in pseudohypha formation (3.26%) compared to the wild-type strain (17.7%). Scanning electron microscopy (SEM) images of wild-type and Δ/Δ*603600 1*/*1* cells further supported the data indicating a decreased amount of pseudohyphae present in the mutant strain (Fig. 5).

10.1128/mSphere.00227-20.4 FIG S4 Difference between single-colony morphologies of CLIB214 and Δ/Δ*603600 1*/*1* strains on Spider medium. Download FIG S4, JPG file, 2.2 MB. Copyright © 2020 Chakraborty et al. 2020 Chakraborty et al. This content is distributed under the terms of the Creative Commons Attribution 4.0 International license.

### Deletion of *CPAR2_603600* decreased biofilm formation.

**FIG 6:**
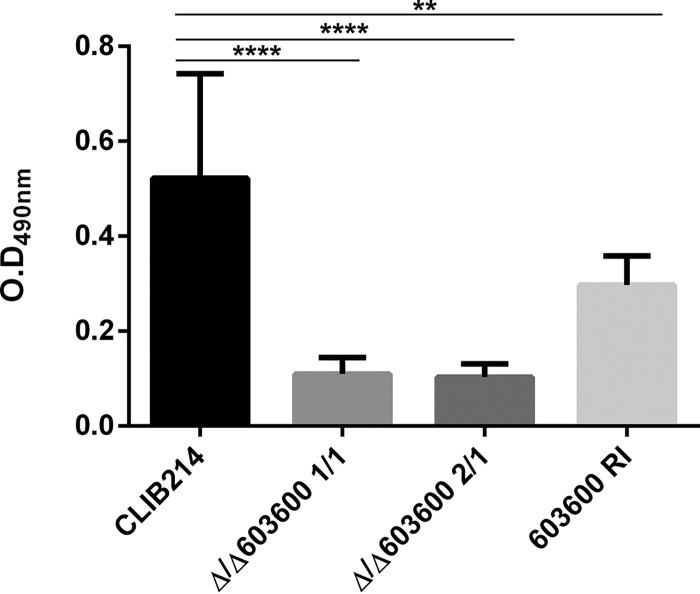
Loss of *CPAR2_603600* results in decreased biofilm formation. The CLIB 214 wild-type, deletion mutant, and reintegrant strains were grown in Spider medium at 37°C for 48 h, and biofilm formation was determined using the XTT cell viability assay. Three independent experiments were performed for biofilm analysis by XTT assay. One-way ANOVA with Dunnett’s *post hoc* test was used to determine statistical significance (**, *P* ≤ 0.01; ****, *P* ≤ 0.0001).

Another major C. parapsilosis virulence factor is the ability to form biofilms on abiotic surfaces such as medical implants. Mature C. parapsilosis biofilms consist of both pseudohyphae and yeast cells. Reductions in the amount of pseudohyphae prompted us to examine if *CPAR2_603600* also affects biofilm formation in this species. Biofilm formation of the wild-type, null mutant, and reintegrant strains was measured using the 2,3-bis-(2-methoxy-4-nitro-5-sulfophenyl)-2H-tetrazolium-5-carboxanilide salt (XTT) metabolic assay. According to our results, the null mutant strains showed a significant decrease in biofilm formation compared to the parental and reintegrant strains (Fig. 6), suggesting that the role of *CPAR2_603600* is not restricted to iron metabolism and filamentous growth regulation.

### Supplementation with additional iron rescued the defective phenotypes of Δ/Δ*603600* mutants.

**FIG 7:**
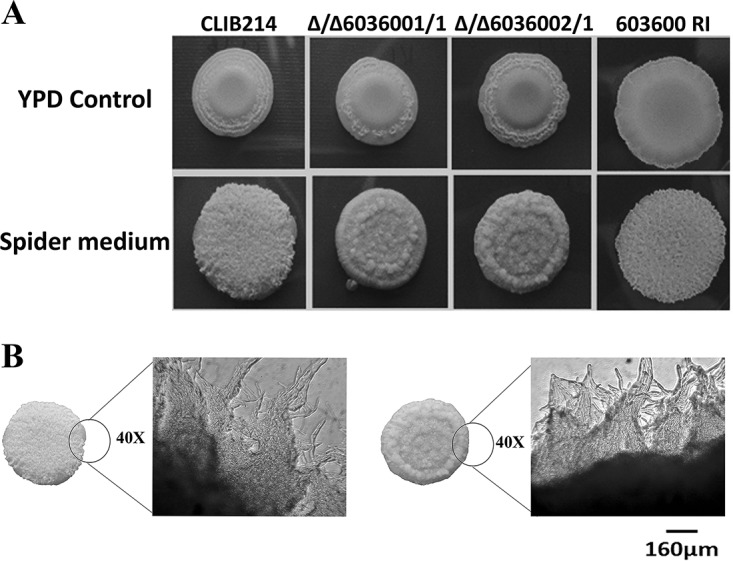
Morphology defect rescued by addition of iron to the preculturing medium. (A) Pseudohypha production was analyzed after growth of each strain overnight at 30°C in 2 mM FeCl_3_-supplemented YPD as a preculturing medium. Cells were then plated on pseudohypha-inducing medium, and images were taken after 7 days of incubation at 37°C. Addition of excess iron to the preculturing medium (YPD) rescued the defective phenotype on solid plates. (B) Microscopic images of the edge of wild-type and Δ/Δ*603600 1*/*1* colonies on solid Spider medium (scale bar, 160 μm).

**FIG 8:**
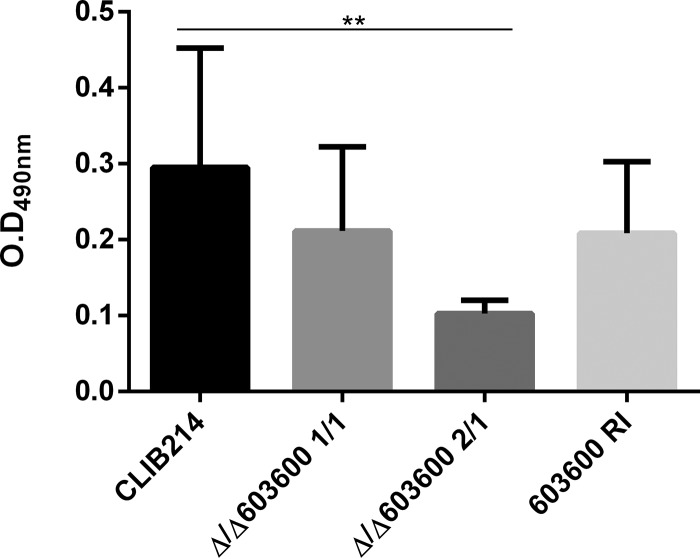
Addition of excess iron to the preculture medium partially rescued the biofilm defect of the deletion mutants. Addition of 2 mM FeCl_3_ to the YPD preculture medium partially rescued the biofilm formation defect. After preculturing of all strains in iron-supplemented YPD, cells were transferred to liquid Spider medium and incubated at 37°C. After 48 h, the XTT assay was applied to measure potential alterations in biofilm formation. Two independent experiments were performed for the biofilm assay. One-way ANOVA with Dunnett’s *post hoc* test was used to determine statistical significance (**, *P* ≤ 0.05).

To examine if the defects in pseudohypha and biofilm formation could be rescued by the reintroduction of iron into the growth medium, all strains were grown in YPD supplemented with 2 mM FeCl_3_ overnight at 30°C, prior to the follow-up examinations. Pseudohypha formation and biofilm formation were examined using methods similar to those described above. Our results revealed that the Δ/Δ*603600* mutant cells regained their ability to form pseudohyphae similarly to the wild-type and reintegrant strains based on colony morphology (Fig. 7). XTT metabolic experiments revealed a partial rescue of the biofilm defect of Δ/Δ*603600* cells by iron supplementation (Fig. 8). Together, these results suggest that supplementation with accessible iron at least partially restores the defective phenotypes gained by *CPAR2_603600* deletion.

### Expression of genes related to iron metabolism in the deletion mutant strains.

**FIG 9:**
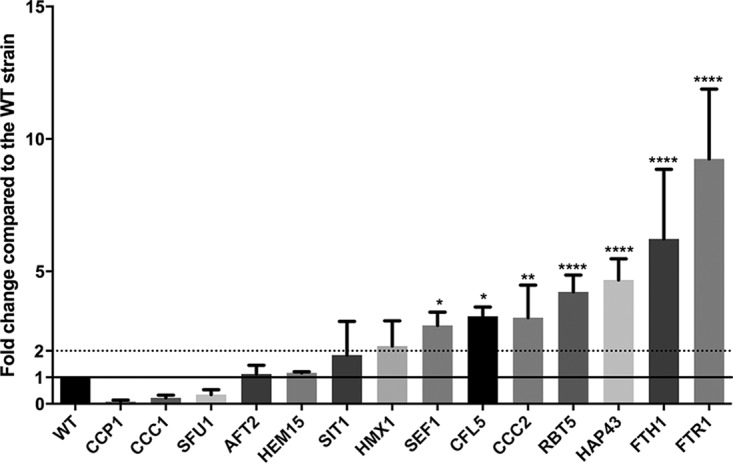
Expression of genes involved in iron metabolism in the homozygous deletion mutant. Expression analysis of 14 genes related to iron metabolism (GO term analysis) was performed using qPCR. The expression levels shown represent results of comparisons to the normalized wild-type (WT) values (WT value = 1; marked by line) for each gene examined. The cells were grown in YPD medium at 30°C for 4 h prior to total RNA isolation. Statistical significance was determined using one-way ANOVA coupled with Dunnett’s *post hoc* test (*, *P* ≤ 0.05; **, *P* ≤ 0.01; ****, *P* ≤ 0.0001).

Next, we examined if the lack of *CPAR2_603600* alters the expression of other genes related to iron metabolism. We selected 14 genes (Table 1) by GO term analysis from the *Candida* genome database, based on their orthologues regulating iron homoeostasis in C. albicans. Using quantitative reverse transcription-PCR (qRT-PCR) analysis, we found that 9 of the 14 analyzed genes showed an increase in expression (average fold change, ≥2) in the Δ/Δ*603600 1*/*1* mutant strain compared to the normalized wild-type values (WT value = 1). Those 9 genes included *RBT5*, *HAP43*, *FTH1*, *FTR1*, *SIT1*, *HMX1*, *SEF1*, *CFL5*, and *CCC2* (Fig. 9). Thus, in the absence of *CPAR2_603600*, altered expression of genes corresponding to ferric reductase, ferrous iron transport, and ferrous iron permease was detected, indicating the presence of a potential compensatory effect.

**TABLE 1:** List of genes related to iron metabolism in C. parapsilosis with their corresponding homologue in C. albicans

CPAR gene ID^*a*^	Candida albicans homologue	Function
CPAR2_210110	*CFL5*	Ferric reductase; induced low iron; ciclopirox olamine, flucytosine induced; amphotericin B, Sfu1 repressed; Tbf1, Hap43 induced
CPAR2_700570	*FTR1*	High-affinity iron permease; required for mouse virulence, low-iron growth; iron, amphotericin B, caspofungin, ciclopirox, Hog1p, Sef1p, Sfu1p, and Hap43p regulated; complements S. cerevisiae *ftr1* iron transport; Hap43p repressed
CPAR2_303120	*CCC2*	Copper-transporting P-type ATPase of Golgi complex; required for wild-type iron assimilation (indirect effect via Fet3p); induced by iron starvation, ciclopirox olamine; caspofungin repressed; not required for virulence in mouse systemic infection
CPAR2_808120	*CFL5*	Ferric reductase; induced in low iron; ciclopirox olamine, flucytosine induced; amphotericin B, Sfu1 repressed; Tbf1, Hap43 induced
CPAR2_405240	*CCC1*	Manganese transporter; required for normal filamentous growth; mRNA binds She3, localized to hyphal tips; repressed by NO, alkaline pH; colony morphology-related regulation by Ssn6; regulated by Sef1, Sfu1, Hap43; Spider biofilm induced
CPAR2_402920	*RBT5*	GPI^*b*^-linked cell wall protein; hemoglobin utilization; Rfg1, Rim101, Tbf1, Fe regulated; Sfu1, Hog1, Tup1, serum, alkaline pH, antifungal drugs, geldamycin repressed; Hap43 induced; required for RPMI 1640 biofilms; Spider biofilm induced
CPAR2_105690	*HMX1*	Heme oxygenase; utilization of hemin iron; transcript induced by heat, low iron, or hemin; repressed by Efg1; induced by low iron; upregulated by Rim101 at pH 8; Hap43-induced; Spider and flow model biofilm induced
CPAR2_700810	*SFU1*	GATA-type transcription factor; regulator of iron-responsive genes; represses iron utilization genes if iron is present; Hap43 repressed; promotes gastrointestinal commensalism in mice; Spider biofilm induced
CPAR2_209090	*HAP43*	CCAAT-binding factor-dependent transcription factor; repressor; also called CAP2; required for low-iron response; similar to bZIP transcription factor AP-1; repressed by Sfu1; ciclopirox olamine induced; rat catheter, Spider biofilm induced
CPAR2_210100	*FTH1*	Protein similar to S. cerevisiae Fth1p, a high-affinity iron transporter for intravacuolar stores of iron; repressed by Sfu1p, amphotericin B, caspofungin; induced by alkaline pH, ciclopirox olamine; regulated by Sef1p, Sfu1p, and Hap43p
CPAR2_801430	*SEF1*	Zn2-Cys6 transcription factor; regulates iron uptake; negatively regulated by Sfu1p, positively regulated by Tbf1; promotes virulence in mice; mutants display decreased colonization of mouse kidneys; Spider biofilm induced
CPAR2_406510	*AFT2*	Putative Aft domain transcription factor; role in regulation of iron metabolism, oxidative stress, adhesion, hyphal growth, colony morphology, virulence; complements S. cerevisiae aft1 mutation; Spider biofilm induced
CPAR2_407560	*SIT1*	Transporter of ferrichrome siderophores, not ferrioxamine B; required for human epithelial cell invasion in vitro, not for mouse systemic infection; regulated by iron, Sfu1, Rfg1, Tup1, Hap43; rat catheter and Spider biofilm induced
CPAR2_102830	*CCP1*	Cytochrome-c peroxidase N terminus; Rim101, alkaline pH repressed; induced in low iron or by macrophage interaction; oxygen-induced activity; regulated by Sef1, Sfu1, and Hap43; Spider biofilm induced; rat catheter biofilm repressed
CPAR2_406320	*HEM15*	Putative ferrochelatase involved in heme biosynthesis; transcript not regulated by iron levels and not affected by a yfh1 null mutation; Spider biofilm repressed

a ID, identifier.

b GPI, glycosylphosphatidylinositol.

### Altered expression of hypha and biofilm regulatory genes in the deletion mutant strain.

**FIG 10:**
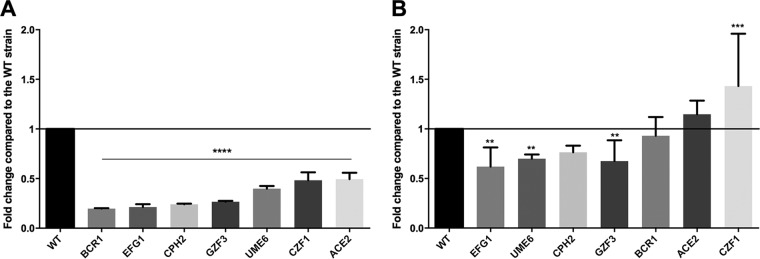
Expression levels of genes involved in pseudohypha and biofilm formation. Genes related to filamentous growth and biofilm formation were examined by qPCR analysis. Expression levels of the selected 7 genes were compared relative to the normalized WT values (WT value = 1). Gene expression analysis performed after growth in Spider medium at 37°C for 24 h (A) and in YPD medium at 30°C for 4 h (B). Statistical significance was determined using one-way ANOVA with Dunnett’s *post hoc* test (**, *P* ≤ 0.01; ***, *P* ≤ 0.001; ****, *P* ≤ 0.0001).

We next analyzed the expression of genes related to biofilm formation and pseudohypha regulation in C. parapsilosis, namely, *EFG1*, *UME6*, *CPH2*, *GZF3*, *BCR1*, *ACE2*, and *CZF1*. Prior to RNA isolation, the wild-type strain and the deletion mutant strain were grown in the filamentous-growth-inducing Spider medium at 37°C (Fig. 10A) and in YPD medium at 30°C (Fig. 10B). Quantitative PCR (qPCR) analysis revealed that even under noninducing conditions, 4 of the 7 examined genes showed a mild decrease in expression relative to the wild-type strain (Fig. 10B). Under filamentous-growth-inducing conditions, all examined pseudohypha and biofilm regulatory genes were markedly downregulated relative to the corresponding genes’ normalized values (set to 1) in the wild-type strain (Fig. 10A).

### Deletion of *CPAR2_603600* in an environmental isolate of C. parapsilosis.

**FIG 11:**
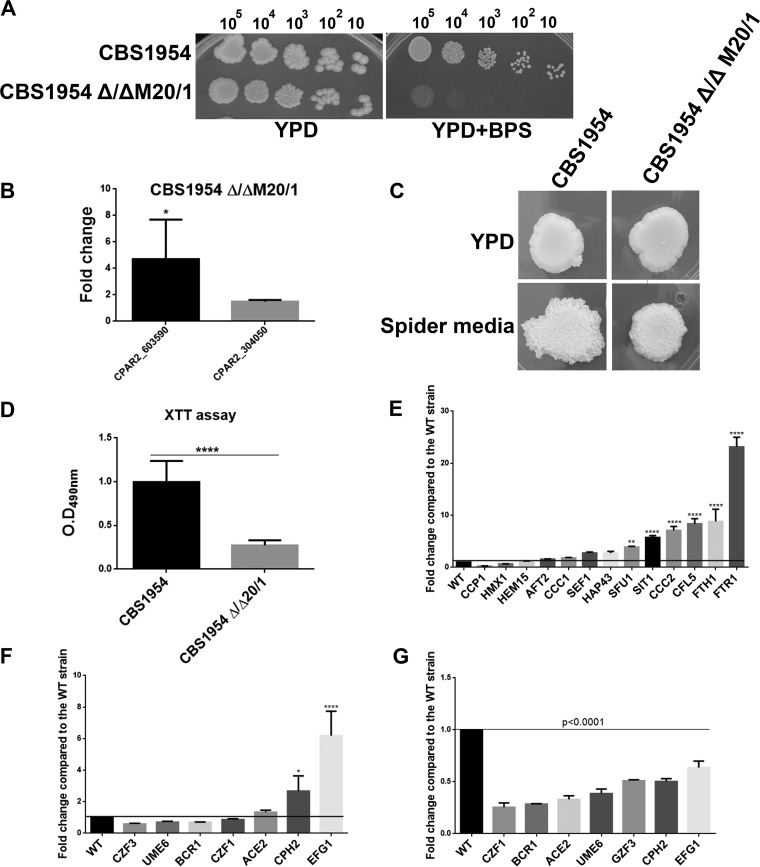
Characterization of *CPAR2_603600*-null mutant in CBS1954 strain. (A) Deletion mutant CBS1954 Δ/ΔM20/1 generated in environmental isolate CBS1954 showed similar growth defects under iron-restricted conditions. (B) Expression of *CPAR2_603590* and *CPAR2_304050* in the absence of *CPAR2_603600* (*, *P* ≤ 0.05). (C) Decrease in filamentous growth on solid Spider medium. (D) Reduction in biofilm formation in the mutant. Statistical significance was calculated using the nonparametric *t* test (****, *P* ≤ 0.0001). (E) Alteration of expression of genes involved in iron metabolism in the homozygous deletion mutant. (F) Expression of biofilm-related genes in YPD medium after growth at 30°C (*, *P* ≤ 0.05; ****, *P* ≤ 0.0001). (G) Expression of biofilm-related genes in Spider medium after growth at 37°C. Statistical significance was calculated using one-way ANOVA with Dunnett’s *post hoc* test.

To examine if *CPAR2_603600* plays a similar role in other C. parapsilosis isolates, a homozygous deletion mutant strain was also generated in the CBS1954 environmental isolate (23) using the SAT1 flipper cassette method (27). We found that the *CBS1954* Δ/Δ*M20*/*1* deletion mutant showed a similar growth defect in the absence of accessible iron (see “YPD +BPS” data in Fig. 11A) and defects in pseudohypha formation (Fig. 11C) as well as in biofilm formation (Fig. 11D). In the generated null mutant strain, the expression levels of both *CPAR2_603590* and *CPAR2_304050* were higher than the level seen in the parental strain (Fig. 11B). Similarly to the Δ/Δ*603600 1*/*1* mutant results, the expression levels of several of the 14 iron metabolism regulatory genes examined were also increased in the *CBS1954* Δ/Δ*M20*/*1* strain (average fold change, ≥2) compared to the normalized wild-type values (CBS1954 or WT = 1) (Fig. 11E). Furthermore, qPCR analysis also revealed that biofilm and pseudohypha regulatory genes were downregulated both under noninducing conditions and under filamentous-growth-inducing conditions relative to the corresponding genes’ normalized values in the wild-type strain, similarly to what was observed in Δ/Δ*603600 1*/*1* (Fig. 11F and G). These observations confirmed that the effect of *CPAR2_603600* gene deletion is strain independent.

## DISCUSSION

Among all the metals, iron plays one of the most important roles in fungal pathogenesis. The availability of free iron is restricted in the blood by the host to provide nutritional immunity. Analyses of the role of metal homoeostasis in virulence of human-pathogenic fungi such as C. albicans, C. neoformans, and A. fumigatus have been reported from numerous previous studies (7). *Candida* spp., being commensal pathogens, evolved different iron uptake mechanisms to survive within different host niches (28). C. albicans utilizes different iron sources from the host through siderophores of other microorganisms or via hemoglobin, transferrin, lactoferrin, and ferritin present within the host. The main pathways involved in iron homoeostasis in C. albicans include surface ferric reductases (*CFL1* and *FRE1* [29] and *CFL95*, *FRE10*, and *RBT2* [29, 30]), multicopper oxidases (*FET3* and *FET99* [12, 31]), and high-affinity iron permeases (*FTR1* [32]). Among the five multicopper oxidase genes present in C. albicans, a mutant with a deletion of the *FET3* gene showed a growth defect under low-iron conditions and a reduction in prostaglandin production (33); however, it remained as virulent as the wild-type strain. Deletion of *FET34* resulted in a slight growth defect under iron-limited conditions and a reduction in hypha formation and showed a hypovirulent phenotype in a mouse model of systemic infection. Disruption of *FET33* resulted in no such defect (25, 34).

In the case of C. parapsilosis, our knowledge of iron homoeostasis is limited. In the present study, we identified three homologues of the S. cerevisiae Fet3p multicopper oxidase in C. parapsilosis by *in silico* analysis. For further analysis, we generated a homozygous mutant strain with a deletion of the gene *CPAR2_603600* and found that the mutant strain product had 79% identity with *CaFET3* and 54% identity with S. cerevisiae *FET3* (*ScFET3*) in amino acid sequence comparison. We found that, similarly to S. cerevisiae and C. albicans, the homozygous null mutant had severe growth defects in iron-limiting medium. We can assume that, as seen with S. cerevisiae, the other two genes are involved in a low-affinity iron uptake system in C. parapsilosis, although further experiments are needed to prove this hypothesis.

In addition to playing a role in iron metabolism, fungal multicopper oxidases can play important roles in developmental processes such as fruiting body formation, pigment formation during asexual development, pathogenesis, and competitor interactions (35). There is also a correlation between iron availability and hypha formation, and the presence of BPS induces expression of the hypha-specific *EFG1* gene in C. albicans (36). Deletion of *FET34* also reduces the level of hypha formation in this fungus. Although, unlike C. albicans, C. parapsilosis is unable to form true hyphae (15), different strains of C. parapsilosis can form pseudohyphae consisting of chain of elongated cells and morphologically distinct biofilms composed of yeast and pseudohyphae (37). The exact mechanism of the yeast-to-pseudohypha switch in C. parapsilosis is poorly understood. Recently, orthologues of C. albicans *EFG1*, *UME6*, *CPH2*, and *CZF1* were shown to play a role in morphology transition in C. parapsilosis (18). Among these, *CZF1* and *UME6* were also shown to be required for biofilm formation. Orthologues of *ACE2*, *BCR1*, and *EFG1* also contribute to biofilm formation in C. parapsilosis (38). It has also been shown previously that various metal ions influence cell differentiation during biofilm maturation in C. albicans and C. tropicalis. In C. albicans, both hypha formation and biofilm formation were inhibited by metallic elements such as arsenic, cadmium, chromium, cobalt, copper, selenium, silver, and zinc and enhanced by lead (39). In the case of C. tropicalis, hypha formation was enhanced by chromium and zinc but inhibited by arsenic, cadmium, cobalt, copper, lead, selenium, and silver (40). Additionally, deletion of the multicopper oxidase gene *CaFET34*, but not deletion of Ca*FET33*, significantly attenuated the filamentous growth of C. albicans (25). In the case of C. parapsilosis, Shafeeq et al. showed Mn^++^-dependent biofilm formation through the activity of the biofilm regulator *BCR1* (41). All these data prompted us to investigate whether a similar effect occurs in C. parapsilosis. We found that the Δ/Δ*603600* mutant showed a visible reduction in pseudohypha formation on solid Spider medium. Analysis of the number of pseudohyphal cells grown in liquid pseudohypha-inducing medium also showed a significant reduction in pseudohypha production. The mutant strain also showed a significant reduction in biofilm formation. But after growth in the presence of excess iron in the medium, the mutants recovered from all phenotypic defects. All the observations described above suggest that *CPAR2_603600* plays a significant role in iron metabolism and morphology transition in C. parapsilosis. Thus, we can assume that the proteins that are involved in morphological switching or biofilm formation in C. parapsilosis might require iron for their normal functioning. As we did not see a complete lack of growth of the mutant under low-iron conditions or a total absence of pseudohypha or biofilm formation, we can assume that the presence of the other two putative multicopper oxidase-encoding genes compensates for the effect of the deletion of one of the genes from the same family. Similarly to the results observed with C. albicans, we found that our mutant showed a significant reduction in prostaglandin production, although the Δ/Δ*603600* mutant was less virulent in a mouse model of systemic infection than C. albicans (19). We also found that in the absence of a *FET3* homologue in C. parapsilosis, several homologues of iron metabolism genes showed higher expression, which can also be a counteracting phenomenon operating to reduce the defect of the lack of a high-affinity iron oxidase in C. parapsilosis. Taken together, our data give a novel insight into the role of a multicopper oxidase gene in iron homoeostasis maintenance, morphology, and biofilm regulation in the human-pathogenic fungus C. parapsilosis. However, further investigation is needed to understand the complete mechanism of iron metabolism in this emerging pathogen.

## MATERIALS AND METHODS

### Strains.

All C. parapsilosis strains used in this study are listed in Table S1 in the supplemental material.

10.1128/mSphere.00227-20.5 TABLE S1 All C. parapsilosis strains used in this study. Download Table S1, XLSX file, 0.01 MB. Copyright © 2020 Chakraborty et al. 2020 Chakraborty et al. This content is distributed under the terms of the Creative Commons Attribution 4.0 International license.

### Primers.

All the primers used in this study are listed in Table S2. Descriptions of the real-time primers for genes involved in pseudohypha and biofilm formation are available in our previous publication (18).

10.1128/mSphere.00227-20.6 TABLE S2 All primers used in this study. Download Table S2, DOCX file, 0.02 MB. Copyright © 2020 Chakraborty et al. 2020 Chakraborty et al. This content is distributed under the terms of the Creative Commons Attribution 4.0 International license.

### Media and growth conditions.

All C. parapsilosis strains were cultivated in YPD (1% dextrose, 1% peptone, and 0.5% yeast extract) at 30°C. Transformants containing the *LEU2* and *HIS1* markers were maintained on Synthetic Complete medium (SC; 2% dextrose, 0.95% yeast nitrogen base, mixture of amino acids, 2% agar) without leucine and histidine. Nourseothricin-resistant colonies were maintained on YPD plates supplemented with 100 μg/ml nourseothricin.

### Construction of deletion mutant strains.

Deletion of *CPAR_6036300* was performed with the fusion PCR method as described in our previous publication (19). For gene deletion in an environmental isolate of C. parapsilosis (CBS1954 strain), the pSFS2SaFET3 plasmid was used. Disruption of the 1,884-bp open reading frame (ORF) was performed as described previously by Horvath et al. (42).

### Gene reintegration.

The DNA construct for reintegration was generated with fusion PCR. The first (2,875-bp) fragment was amplified using the primer pair UpFP and ORF-UP-RP-Fus (see file S2 in the supplemental material) and consisted of 1,000 bp upstream of the ORF and the ORF without the stop codon. The second (3,151-bp) mCherry-NAT fragment was amplified using mCherry-FP-Fus and mCherry-RP-Fus with pMG2343 plasmid (43) as the template. The third and final (948-bp) downstream fragment was amplified using the primer pair ORF-DN-Fp-Fus and ORF-DN-RP. After purification of all the three fragments, fusion PCR was performed to join all three fragments to generate the final 6,974-bp DNA construct. After gel purification, this DNA construct was used to transform the Δ/Δ*603600 2/1* strain and the transformants were selected on a YPD plate containing 100 μg/ml nourseothricin. The transformants were confirmed by Southern blotting, and expression of the gene was also verified by qRT-PCR analysis.

### Phenotypic characterization.

Growth of the mutant strains was examined under various conditions, designed to identify nutritional, cell wall, and osmotic- and oxidative-stressor-responsive phenotypes (18). All deletion mutant strains were grown in 2 ml of YPD medium prior to each experiment at 30°C overnight. The cultures were then washed three times with 1× phosphate-buffered saline (PBS) and diluted in 1× PBS after the desired cell concentrations were determined. A 5-μl volume from each dilution was plated on various plates followed by incubation for 2 to 3 days at 30°C.

### Pseudohypha formation on solid plates.

After the desired cell concentration (optical density [OD] of 0.08) was set, cells were plated on YPD medium and pseudohypha-inducing Spider medium (1% peptone, 1% yeast extract, 1% mannitol, 0.5% NaCl, 0.2% K_2_HPO_4_) (44) and incubated at 37°C for 7 days.

### Morphology analysis by fluorescence-activated cell sorter (FACS) analysis.

Cells were collected from overnight cultures (37°C, 5% CO_2_) from YPD and Spider media and suspended in 1 ml of 4% paraformaldehyde followed by 30 min of incubation at room temperature, with continuous rotation. Following incubation, cells were washed four times with 1× PBS, and pellets were suspended in 0.5 ml 1% bovine serum albumin (BSA) (Sigma-Aldrich), followed by incubation at room temperature for additional 30 min with rotation. The cells were then washed three times with 1× PBS and suspended in 200 to 400 μl of the same buffer, depending on the cell concentration (approximately 10^8^/ml cell). Then, 100 μl of the suspension was transferred into a new Eppendorf tube and the morphology was analyzed by the use of Amnis Flowsight and the pseudohyphae were quantified by the use of IDEAS software (Amnis).

### Biofilm formation.

Biofilm formation was examined in liquid Spider medium by analysis of growth in 96-well polystyrene plates. All C. parapsilosis strains were grown overnight in YPD at 30°C and were then diluted to an optical density at 600 nm (OD_600_) of 0.5 in 2 ml of Spider medium. The 96-well flat bottom microtiter plates were pretreated overnight with 10% FBS and then washed 2 times with 1× PBS before addition of 100 μl of cell suspension (5 × 10^5^ cells) from the Spider medium. Cells were incubated at 37°C for 90 min at 180 rpm for initial adherence of the biofilm. The wells were then washed 2 times with 1× PBS, and 100 μl of fresh Spider medium was added to each well. The plates were kept at 37°C for 48 h before the quantification.

### XTT reduction assay.

Biofilm formation was quantified using the XTT reduction assay (45). Briefly, 0.5 mg/ml XTT solution in PBS was prepared, filter sterilized, and stored at –70°C. Menadione solution (10 mM) in acetone was prepared freshly immediately before use. Prior to the assay, 1 μl of menadione solution was added to 10 ml of PBS. First, the biofilms were washed 3 times with 1× PBS and then 200 μl of XTT-menadione solution (ratio of 5 to 1 by volume) was added to each well. The plate was then incubated at 37°C for 2 h in the dark. After incubation, 100 μl of each solution was transferred to another 96-well plate and measured with a microtiter plate reader at 490 nm. Ten technical parallels were used per strain, and cell-free Spider medium was used as a control. The values of the control wells were subtracted from the test absorbance values to reduce the background interference. Three independent experiments were performed for the assay.

### RNA extraction and reverse transcription-PCR.

For RNA extraction, all C. parapsilosis strains were grown overnight in 2 ml of YPD medium at 30°C with shaking applied at 180 rpm. The next day, the cells were freshly inoculated in 5 ml of YPD or YPD supplemented with 100 μM/ml BPS (OD of 0.2). For gene expression analysis, strains were grown overnight in 2 ml YPD medium at 30°C with shaking applied at 180 rpm, followed by washing steps performed with 1× PBS, and were reinoculated in liquid Spider medium for incubation at 37°C for 24 h. RNA was isolated using a RiboPure yeast RNA isolation kit (Ambion) following the manufacturer’s instruction. A total of 500 ng RNA was used for cDNA synthesis. cDNA was synthesized using a RevertAid First Strand cDNA synthesis kit (Thermo Scientific) according to the manufacturer’s instruction.

### Real-time PCR.

Real-time PCR was carried out in a final volume of 20 μl using Maxima SYBR green/fluorescein qPCR Master Mix (Thermo Scientific) (2×). The reaction was performed in a C1000 thermal cycler (Bio-Rad) using the following reaction conditions: 95°C for 3 min, 95°C for 10 s, 60°C for 30 s, and 65°C for 5 s for 50 cycles. Fold change in mRNA expression was calculated by the threshold cycle (ΔΔ*C_T_*) method (real-time PCR applications guide; Bio-Rad). *TUB4* was used as a housekeeping gene as an internal control.

### Statistical analysis.

One-way analysis of variance (ANOVA) was used to determine the differences in biofilm formation by different strains. Differences were considered statistically significant at *P* values of ≤0.05 (*, *P* ≤ 0.05; **, *P* ≤ 0.01; ***, *P* ≤ 0.001; ****, *P* ≤ 0.0001).
