# Supplementary figures and images for: Iron Metabolism, Pseudohypha Production, and Biofilm Formation through a Multicopper Oxidase in the Human-Pathogenic Fungus Candida parapsilosis
Source: mSphere. 2020 May 13;5(3):e00227-20. doi: 10.1128/mSphere.00227-20 (PMC7227767; doi:10.1128/mSphere.00227-20)

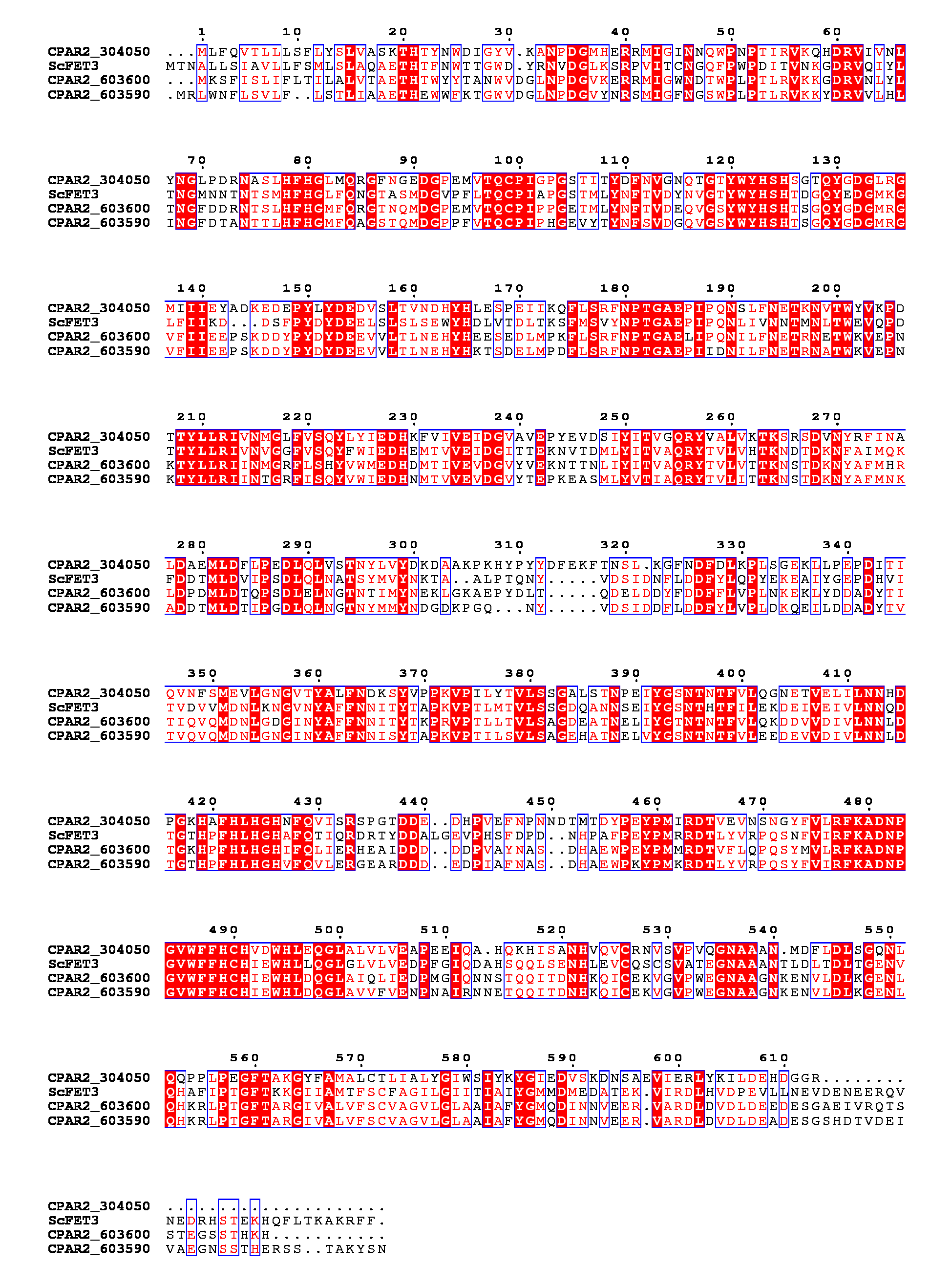

Supplement: FIG S1 [file mSphere.00227-20-sf001.tif]

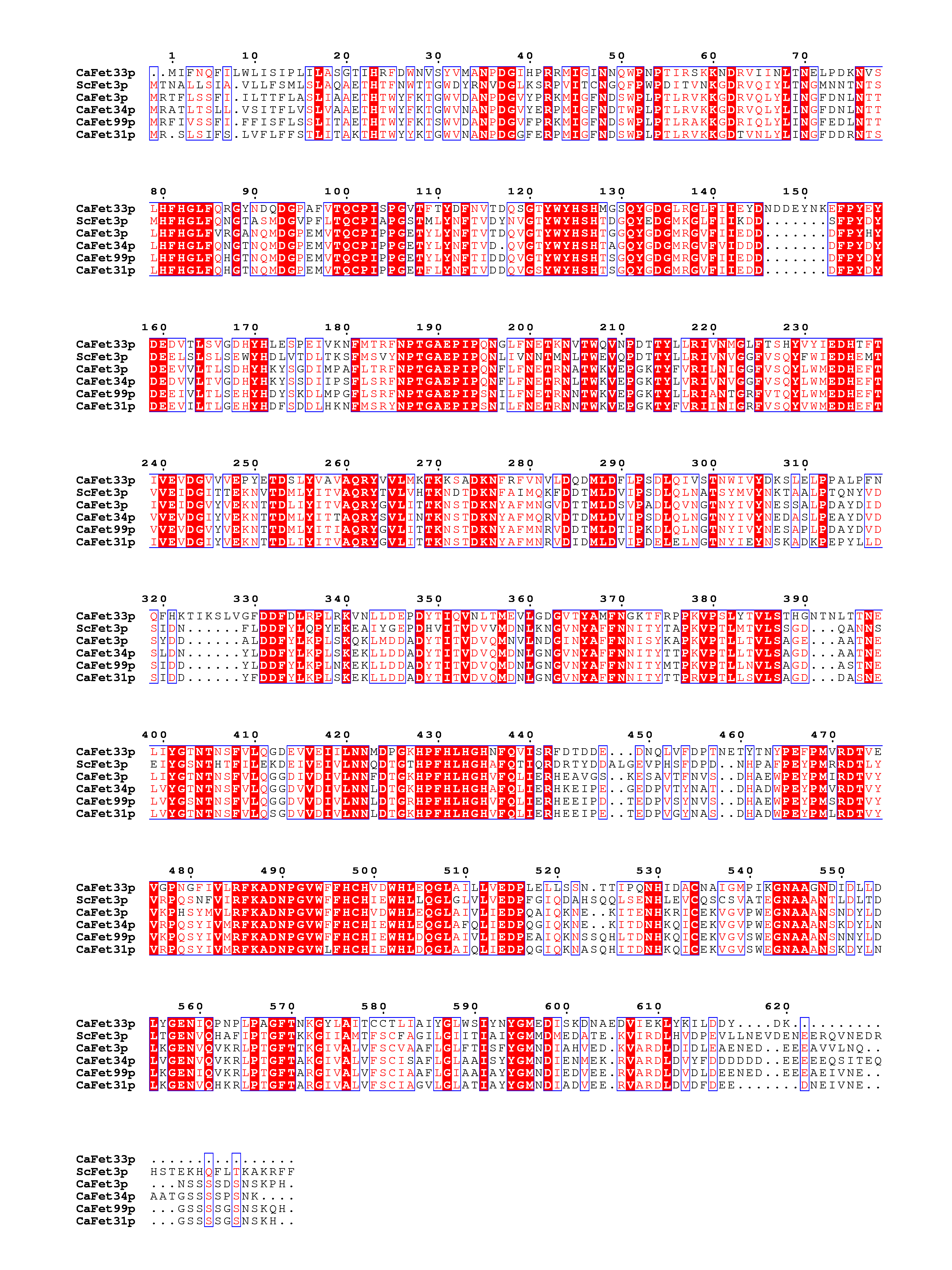

Supplement: FIG S2 [file mSphere.00227-20-sf002.tif]

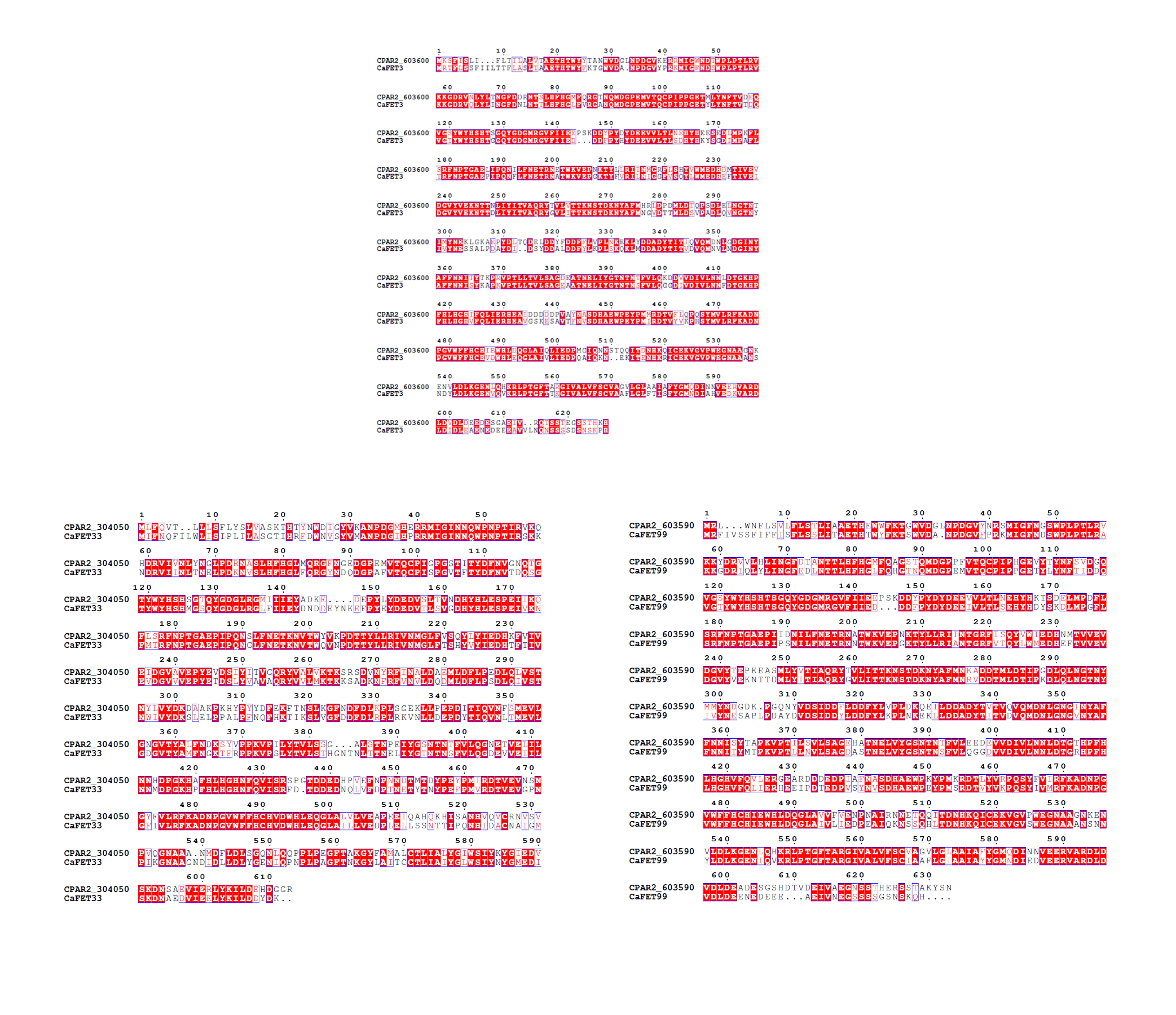

Supplement: FIG S3 [file mSphere.00227-20-sf003.tif]
