# Supplementary material for: Iron Metabolism, Pseudohypha Production, and Biofilm Formation through a Multicopper Oxidase in the Human-Pathogenic Fungus Candida parapsilosis
Source: mSphere. 2020 May 13;5(3):e00227-20. doi: 10.1128/mSphere.00227-20 (PMC7227767; doi:10.1128/mSphere.00227-20)
Supplement: TABLE S2 [file mSphere.00227-20-st002.docx]

| **Primer Name** | **Sequence (5' to 3')** |
| --- | --- |
| 603600 RTF | AGTAGGTGTGCCATGGGAAG |
| 603600 RTR | GTGAATCCAGTGGGCAATCT |
| qTub4F | GAACACTTATGCCGAGGACAAC |
| qTub4R | ACTCTCACCACTGACTCCTTGC |
| 603600 Pri1 | GCTTTCCAATGTCGTAGTTTACC |
| 603600 Pri3 | CACGGCGCGCCTAGCAGCGGCGAGTGCCAATATAGTGAGG |
| 603600 Pri5 | GCAGGGATGCGGCCGCTGACACGAGGTCTTGCATCTACTTAGCTCGGATCCACTAGTAACG |
| 603600 Pri4 | GTCAGCGGCCGCATCCCTGCGCCAGAGACTTGGATGTTG |
| 603600 Pri6 | GCGAAATAGCACTCCCATTATC |
| 210110 RTFP (CFL5) | GTTTTTATTCCCCGGCCTTAC |
| 210110 RTRP(CFL5) | TTGCTCTTGCAGCTTCTTCA |
| 700570 RTFP(FTR1) | GGTGCTTCTTGCCTTTTTGA |
| 700570 RTRP(FTR1) | CTAGAATTGCCCCAAACCAA |
| 303120 RTRP(CCC2) | TGAAGAAATGGCTGTTGGTG |
| 303120 RTFP(CCC2) | GACATCGTTTTCCAAAGCATCA |
| 405240 RTFP (CCC1) | CCTTCCATACGGTTCCACAG |
| 405240 RTRP(CCC1) | TGATGCTATTGAGGCTGCTG |
| 402920 RTRP (RBT5) | GGCGAAGCAATTGGTAGTGT |
| 402920 RTFP (RBT5) | GCCAAAGCTTGTGCAACAG |
| 105690 RTFP (HMX1) | GGAAACCAGAAATTGCCAGA |
| 105690 RTRP (HMX1) | TGAGACATTTTGGGGCTGA |
| 700810 RTFP (SFU1) | TGAAGCGACCACCAAATACA |
| 700810 RTRP (SFU1) | GCTGATCCACCAGTTCCATT |
| 209090 RTFP (HAP43) | CGTCGTATTCGCAACTTTCAC |
| 209090 RTRP(HAP43) | TGGGTTCAGAACAGCATTGA |
| 210100 RTFP (FTH1) | GGGAAGGGACTTTTTCCATC |
| 210100 RTRP(FTH1) | CTCCCCAATTTGACTTTCCA |
| 801430 RTFP (SEF1) | GTCACCAATCCCAGGACAAC |
| 801430 RTRP(SEF1) | TCAAGAATAGGGGTGGCTTG |
| 406510 RTFP (AFT2) | TGAACCCTTCGCATATCCTC |
| 406510 RTRP(AFT2) | GGAATTCCCATTGGTCTGTG |
| 407560 RTFP(SIT1) | GGCGTTTTTGGTTGGTTATG |
| 407560 RTRP(SIT1) | GCAGTGATGGCATTGACAGT |
| 102830 RTFP (CCP1) | ATACGCCGCTGCTGATAACT |
| 102830 RTRP(CCP1) | TGCAGTGCCTTTATTTTGGA |
| 406320 RTFP(HEM15) | TCCGGTTTAGCTGATCTCGT |
| 406320 RTRP(HEM15) | ATGTACCTGTGGCCCTTTCA |
| Univ_Primer_2 | CCGCTGCTAGGCGCGCCGTGACCAGTGTGATGGATATCTGC |
| HIS Chk1 | AAAATCAATGGGCATTCTCG |
| HIS Chk2 | TGGGAAGCAGACATTCAACA |
| LEU2 Chk1 | GAAGTTGGTGACGCGATTGT |
| LEU2Chk2 | TTCCCCTTCAATGTATGCAA |
| ORF UP RP Fusion | ATGTTTATGAGTAGATGAGCCTTCAGTTG |
| mCherry FP Fusion | CAACTGAAGGCTCATCTACTATGGTTTCAAAAGGTGAAGA |
| mCherry RP Fusion | ACTGGATGGCGGCGTTAGTATCGAATCG |
| ORF DN FP Fusion | CGATTCGATACTAACGCCGCGGAGCTTACTTTTTGTTACG |
| ORF DN RP | CGTATTCATCTTTCATATGTTGTCG |
| CPAR2_304050 ReTi FP | GATCATTATCACTTGGAGTCGC |
| CPAR2_304050 ReTi RP | GTGGTATTGGTTCAGCACC |
| CPAR2_603590 ReTi FP | GATGGTGTATACACCGAACCAA |
| CPAR2_603590 ReTi RP | GTCAGTGGAGTTTTTAGTGGTG |
| UpFP | ATGCGGGCCCCTTTCCAATGTCGTAGTTTACC |
| UpRP | CCGCTCGAGTGTTGGATACACTACTCTAGATC |
| DsFP | TCCCCGCGGGGAGCTTACTTTTTGTTACGA |
| DsRP | CGAGCTCGACACAGTGCCATATATACACC |
